# Analyzing Best Practices for Pediatric Well-Child Clinic Visits in the United States for Children Aged Three to Five Years: A Review

**DOI:** 10.7759/cureus.45194

**Published:** 2023-09-13

**Authors:** Okelue E Okobi, Patience F Akahara, Onyinyechukwu B Nwachukwu, Thelma O Egbuchua, Olamide O Ajayi, Kelechukwu P Oranu, Ifreke U Ibanga

**Affiliations:** 1 Family Medicine, Larkin Community Hospital Palm Springs Campus, Miami, USA; 2 Family Medicine, Medficient Health Systems, Laurel, USA; 3 Family Medicine, Lakeside Medical Center, Belle Glade, USA; 4 Family Medicine, Inglewood Medical Centre, Edmonton, CAN; 5 Neurosciences and Psychology, California Institute of Behavioral Neurosciences & Psychology, Fairfield, USA; 6 Family Medicine, American International School of Medicine Georgetown, Guyana, USA; 7 Pediatrics and Neonatology, Delta State University Teaching Hospital, Oghara, NGA; 8 Internal Medicine, Obafemi Awolowo College of Health Sciences, Olabisi Onabanjo University, Sagamu, NGA; 9 Obstetrics and Gynecology, Kenechukwu Specialist Hospital and Maternity Enugu, Enugu, NGA; 10 Pediatrics, Thompson General Hospital, Manitoba, CAN

**Keywords:** chronic illnesses, parental involvement, anticipatory guidance, vaccination, growth monitoring, developmental milestones, preventive healthcare, children aged 3-5 years, pediatric care, well-child visits

## Abstract

Inadequate routine healthcare check-up visits for children aged three to five years impose substantial economic and social burdens due to morbidity and mortality. The absence of regular well-child visits and vaccinations leads to avoidable diseases, underscoring the need for a renewed emphasis on childhood immunizations and check-ups. Out of 160 articles initially screened after removing duplicates, 45 were chosen for full-text review following initial title and abstract screening by two independent reviewers. Afterward, 20 studies met the predefined inclusion criteria during the final assessment of full-text articles, and data were systematically extracted from these selected studies using standardized forms to ensure accuracy and consistency. Well-child visits promote holistic development, health, and well-being in children aged three to five years. Following established guidelines and evidence-based practices, healthcare professionals provide assessments, vaccinations, and guidance for a healthy future. Despite challenges, well-child visits are vital for preventive care, empowering informed decisions for children's growth and development. The benefits of well-child visits encompass growth monitoring, anticipatory guidance, and preventive measures, crucial for children with chronic illnesses. Key components include comprehensive assessments, developmental screenings, vision and hearing evaluations, immunizations, health education, and counseling. In the case of juvenile diabetes, parental education is paramount. Parents need to understand the intricacies of insulin administration, including proper dosage calculation based on glucose measurements, meal planning, and the importance of timing insulin injections. Implementing guidelines and principles by organizations such as Bright Futures and the American Academy of Pediatrics ensures holistic care, parent involvement, and evidence-based practices. This review explores best practices and guidelines for such visits, emphasizing their role in monitoring and promoting children's development.

## Introduction and background

Pediatric-associated morbidity and mortality resulting from inadequate routine healthcare check-up visits impose a considerable national economic and social burden worldwide, including in the United States. The data collected from medical check-up records from January 2019 to December 2020 revealed that the risk of disease-related adverse outcomes in a population is higher if routine non-healthcare check-up visits are ignored [1-4]. For example, missed vaccinations have been identified as a significant contributor to the rise of pediatric vaccine-preventable illnesses in the United States. According to the Centers for Disease Control and Prevention (CDC), millions of children in the United States are under-vaccinated or unvaccinated against preventable diseases [1-5]. The CDC website provides a recommended immunization schedule for children from birth through 18 years of age, and parents can check with their child's healthcare provider to ensure that their child is up to date on vaccinations [1-3]. However, missed well-child visits (WCVs) have resulted in significant declines in vaccination coverage in children at all milestone ages [1]. The decline in vaccination coverage has markedly increased the risk of vaccine-preventable diseases in children, including measles, polio, and pertussis [1,2]. Re-prioritizing childhood immunizations and well-visits can prevent the re-emergence of vaccine-preventable diseases, and it is essential that parents and care providers prioritize children's well-child schedule to prevent the rise of pediatric-preventable illnesses in the United States. In addition to that, re-prioritizing childhood immunizations has a far-reaching impact beyond the prevention of vaccine-preventable diseases. This, in turn, can help prevent non-vaccine-preventable diseases by alleviating the burden on healthcare systems, reducing the risk of hospital-acquired infections, and improving overall population health [1-5].

Also, these absences of regular and full body healthcare check-ups for children aged three to five years can lead to undetected health issues, delayed interventions, and potentially fatal consequences [2-6]. This translates into increased medical costs, reduced productivity, and an emotional toll on families. The economic impact encompasses direct medical expenses for treating preventable illnesses, emergency care, and hospitalization, often straining healthcare resources. Moreover, the long-term consequences of inadequate health supervision during these critical developmental years can lead to reduced educational attainment, hindered workforce participation, and a perpetuated cycle of health disparities [5-8]. As a result, investing in and promoting routine healthcare check-up visits for pediatric populations is essential not only for safeguarding the well-being of the younger generation but also for alleviating the economic and social ramifications that arise from preventable disease burdens and loss of life [1-9].

Pediatric WCVs are marked by significant changes in speech and language proficiency, as well as the refinement of fine and gross motor skills. Their burgeoning social interactions and cognitive abilities further underscore the importance of this developmental phase. Regular WCVs during this stage assume a vital role in assessing a child's progress, offering a valuable chance to identify any potential deviations from the expected trajectory and intervene promptly if necessary [8-10].

Moreover, the preschool years witness the emergence of emotional and behavioral skills in children, a domain equally crucial to their holistic development. Within this context, WCVs serve as a platform for healthcare providers to engage with parents, addressing concerns related to behavior, furnishing strategies for managing behavioral obstacles, and discerning subtle signs of emotional or psychological challenges that might warrant attention. Adding to the array of advantages inherent in these WCVs, it is noteworthy that this age range coincides with a critical juncture for immunizations. The routine check-ups between three and five years of age routinely encompass pivotal immunizations that bolster a child's immunity, safeguarding them against a spectrum of severe diseases, and forging a shield of protection that will serve them well into the future [3-6,11-14].

This comprehensive review analyzes the best practices and guidelines for pediatric WCVs in children aged three to five years. We will explore the importance and benefits of these visits and the components that make up a successful visit. Additionally, we will examine the best practices and potential challenges associated with implementing them and review the guidelines and principles that should be followed during these visits. By doing so, we hope to provide a thorough understanding of the importance of these visits and the relationship between continuity, quality of care, and long-term health outcomes that can guide healthcare providers in delivering the most effective care to pediatric patients, ultimately leading to improved health and well-being in the years to come.

Methods and Materials, and Literature Search

**Figure 1:**
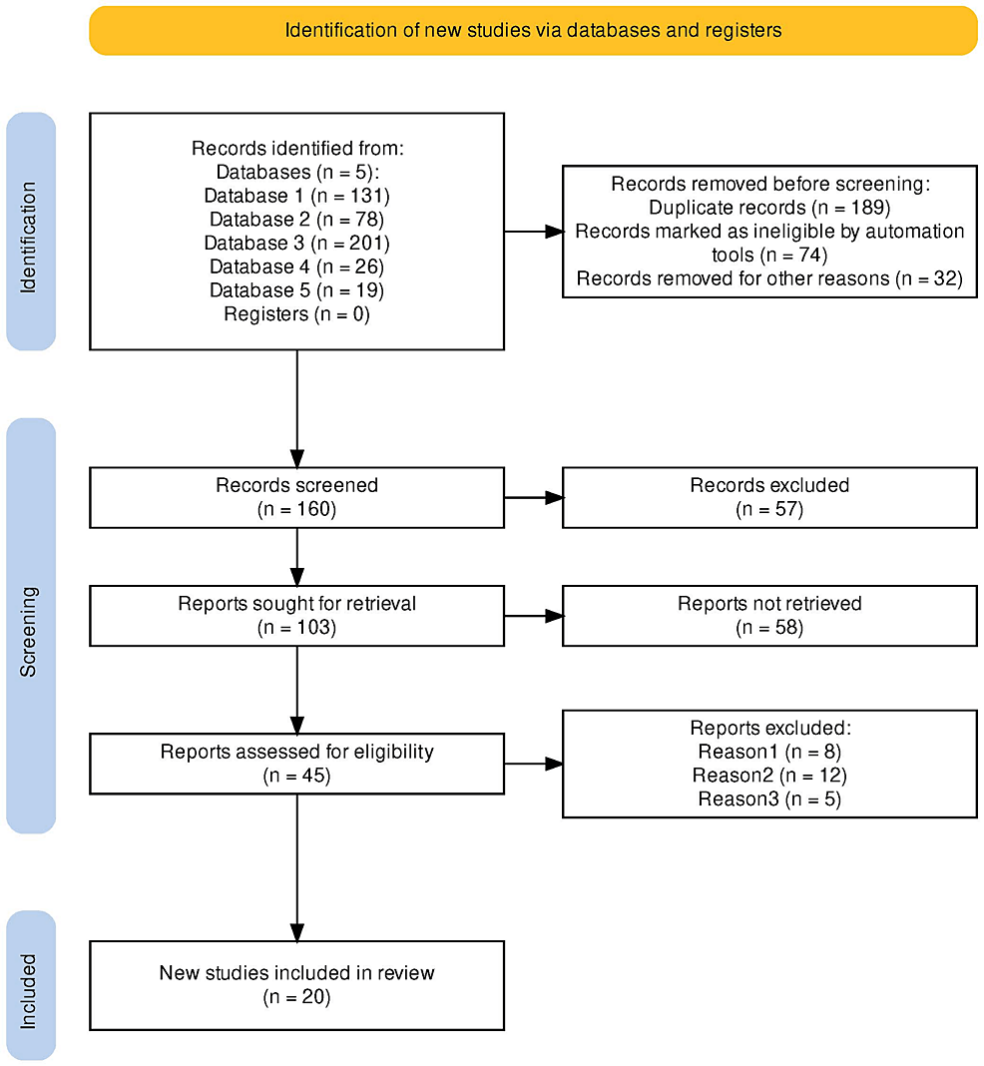
PRISMA flow diagram indicating the article selection process and criteria for the articles used in this systematic review. PRISMA, Preferred Reporting Items for Systematic Reviews and Meta-Analyses

For the present systematic review, an in-depth and comprehensive literature search was conducted on various online databases, including PubMed, Science Direct, Google Scholar, Embase, and Cochrane Library, for peer-reviewed and English-language articles published between 2000 and 2023. The search used various combinations of MeSh terms: “Well child visit,” “well child clinic,” “pediatric healthcare check-up visit,” and “well-baby care,” “preventive care,” and “primary care,” and Boolean operators (AND, OR, NOT) and parentheses to specify various combinations of operations in PubMed, Science Direct, Google Scholar, Embase, Cochrane Library, and HINARI: (1) PubMed (All terms with OR): (“Well child visit” OR “well child clinic” OR “pediatric healthcare check-up visit” OR “well-baby care”) AND (“preventive care” OR “primary care”); (2) Science Direct (Well-child visit and preventive care): (“Well-child visit” OR “pediatric healthcare check-up visit” OR “well-baby care”) AND “preventive care”; (3) Google Scholar (primary care and well-child visit): (Well child visit” OR “pediatric healthcare check-up visit” OR “well-baby care”) AND “primary care”; (4) Embase (Well-child visit and NOT primary care): (“Well child visit” OR “pediatric healthcare check-up visit” OR “well-baby care”) NOT “primary care”; (5) Cochrane Library (Well-child visit OR well-child clinic): (“Well child visit” OR “well-child clinic”) AND (“preventive care” OR “primary care”). We also used exact phrase search, and these combinations allowed us to specify different search criteria related to child healthcare visits, preventive care, and primary care. The Preferred Reporting Items for Systematic Reviews and Meta-Analyses (PRISMA; Figure 1) was then used as the preferred guideline for consistency. To locate the articles, we employed keywords that included “well child clinic,” “pediatric healthcare check-up visit,” and “well-baby care,” alongside MeSH terms “preventive care” and “primary care.” The references of the included studies were searched to identify additional articles. The articles sought for were mainly those that analyzed the best practices related to well-child clinic visits, those that explored the significance of such clinical visits, and those that reviewed the components making up successful and effective well-child clinic visit, as well as the best practices and challenges linked to execution of best practices guidelines and principles. The present systematic review has focused on the best practices and guidelines for well-child clinic visit for children aged three to five years. To attain the objectives, the interventions are to be practice-based and applicable to well-child clinic care delivery.

Inclusion and exclusion criteria

For the current systematic review, we excluded studies that had focused on the evaluation of the process of quality improvement in best practices in well-child clinic visits without acknowledging specific practice changes to child care delivery; articles that focused on one topic in well-child care as opposed to tackling the different aspects of well-child clinic practices more generally; articles that were published before 2010 and those that were published in languages other than English, and studies that focused on tackling changes in well-child clinic without tackling the issue of changes in service delivery.

A total of 160 articles were screened for primary screening (title and abstract review) after removing duplicates. Two independent reviewers conducted the initial screening of titles and abstracts to identify potentially relevant articles. After the initial screening, a total of 45 studies were included for secondary screening or full-text review. Full-text articles were obtained for studies that met the initial screening criteria or where there was uncertainty. The full-text articles were then assessed for final inclusion based on the predefined inclusion and exclusion criteria, and, finally, 20 studies were included in this review for data extraction. Data were systematically extracted from the selected studies and assessed using standardized forms to ensure consistency and accuracy.

Consequently, the studies included in this review (Table 1) were observational studies, randomized controlled trials, and systematic reviews, which included child participants aged zero to five years (with focus on three to five years), and whose findings are directly related to delivery and reception of well-child clinic services, care quality, and child health and development outcomes. We also independently screened the studies and titles with the objective of excluding articles that were duplicated and were irrelevant to the study objectives. The abstracts of studies were also screened using a brief and structured screening tool with the objective of establishing if the selected article satisfied the inclusion criteria, including the topic being studied, the study design, target population, sample size, and study location. We also reviewed the abstract screening outcomes, even as disagreements were solved through general consensus. For the accepted abstracts, retrieval of full texts was conducted, even as a structured form was employed in the extraction of data pertaining to the study design, methodology, results, and findings.

**Table 1:** The systematic review includes a list of studies reviewed, including the author name, study title, study objective, research design, sample size, year of publication, and publishing journal. EPDS, Edinburgh Postnatal Depression Scale; PPD, postpartum depression; PRO, patient-reported outcome; RCT, randomized controlled trial; WCC, well-child clinic; WCV, well-child visit

Author	Title	Study objectives	Research design	Sample size	Publication year	Journal
Turner [6]	Well-child visits for infants and young children	To assess the impacts of the comprehensive assessment of a child in WCC and also the opportunity for further evaluation if abnormalities are detected	Literature review	21 studies	2018	American Family Physician
Nunes and Ayala [7]	Communication techniques used by pediatricians during well-child program visits: a pilot study	To analyze occurrences during well-child program visits, specifically the communication techniques used by pediatricians.	Mixed methods research	49 visits to five pediatricians	2010	Patient Education and Counseling
Bailey-Davis et al [8]	Comparing enhancements to well-child visits in the prevention of obesity: ENCIRCLE cluster-randomized controlled trial	To test the comparative effectiveness of standard care WCVs in relation to two enhancements: adding a PRO measure (PRO WCV) and PRO WCV plus food care	RCT	2,025 parents and their preschool-aged children	2022	MC Public Health
Garg et al. [9]	Improving the management of family psychosocial problems at low-income children's well-child care visits: the WE CARE Project	To evaluate the feasibility and impact of an intervention on the management of family psychosocial topics at well-child care visits at a medical home for low-income children	RCT	200 parents and 45 residents		Pediatrics
Coker et al. [10]	Well-child care clinical practice redesign for young children: a systematic review of strategies and tools	To conduct a systematic review on WCC clinical practice redesign for children aged 0 to 5 years	Systematic review	33 studies	2013	Pediatrics
Peetoom et al. [11]	Does well-child care education improve consultations and medication management for childhood fever and common infections? A systematic review	To review the effect of providing educational interventions about childhood fever and common infections in WCCs), prior to illness episodes, on parental practices: healthcare-seeking behavior and medication management	Systematic review	Eight studies	2017	Archives of Disease in Childhood
Moyer and Butler [12]	Gaps in the evidence for well-child care: a challenge to our profession	To review the evidence for the effectiveness of interventions that include health supervision (well-child care) and recommendations for office-based preventive interventions	Mixed methods research	Seven organizations	2004	Pediatrics
Olson et al. [13]	Overview of the content of health supervision for young children: reports from parents and pediatricians	To describe the content of anticipatory guidance provided to parents of infants and toddlers and to identify primary areas of unmet needs as reported by both parents and pediatricians	Mixed methods research	2,068 parents	2004	Pediatrics
McDermott et al. [14]	Overview of pediatric emergency department visits	To provide an overview of emergency department child visits	Literature review	N/A	2015	europepmc.org
Bailey‐Davis [15]	Feasibility of enhancing well-child visits with family nutrition and physical activity risk assessment on body mass index	To evaluate the feasibility and impact of integration of behavioral risk assessment into WCVs as recommended by clinical guidelines	Quasi-experimental study	10,647 children	2019	Obesity Science & Practice
Frayne et al. [16]	Interconception care for mothers at well child visits after implementation of the IMPLICIT model.	To review the effects of interconception care for mothers at WCVs following the implementation of the IMPLICIT model	Mixed method research	307 mothers	2021	Maternal and Child Health Journal
Emerson et al. [17]	Postpartum depression screening for new mothers at well child visits	To determine prevalence of mothers who scored in the at-risk range using the EPDS at each of the 2-, 4-, and 6-month WCVs in a pediatric outpatient practice and to examine feasibility factors relative to extending the current standard of care for PPD screening	Prospective cohort study	43 postpartum women	2018	MCN: The American Journal of Maternal/Child Nursing
Chaudron et al. [18]	Legal and ethical considerations: risks and benefits of postpartum depression screening at well-child visits	To review the ethical and legal considerations of and outline the risks of screening or not screening for PPD at pediatric visits	Literature review	57 studies	2007	Pediatrics
Schor [19]	Rethinking well-child care	To review the existing state of well-child care and make recommendations for improvement	Literature review	129 studies	2004	Pediatrics
Ragavan et al. [20]	Parents' perspectives about discussing climate change during well-child visits	To examine parents’ perspective during WCVs	Cross-sectional study	371 parents	2021	The Journal of Climate Change and Health
Kuo et al. [21]	Rethinking well-child care in the United States: an international comparison	To describe the process of well-child care delivery in industrialized nations and compare it to the U.S. model of child healthcare	Literature review	93 studies	2006	Pediatrics
Tanner et al. [22]	Reflections on well-child care practice: a national study of pediatric clinicians	To assess perspectives about the practice of well-child care among pediatric clinicians, especially in the areas of child development and behavior	Mixed method research	282 pediatricians and 41 pediatric nurse practitioners	2009	Pediatrics
Halfon et al. [23]	Duration of a well-child visit: association with content, family-centeredness, and satisfaction	To examine the length of WCVs and the associations of visit length with content, family-centered care, and parent satisfaction among a national sample of children	Cross-sectional telephone survey	2,068 parents of children aged 4 to 35 months	2011	Pediatrics
Norlin et al. [24]	Delivery of well-child care: a look inside the door	To describe the delivery of well-child care and its components, to compare that delivery with recommendations in Bright Futures, and to compare delivery of well-child care for children with special healthcare needs with that for children without special needs	Observational research method	43 pediatricians and 9 midlevel providers	2011	Academic Pediatrics
Abdus and Selden [25]	Adherence with recommended well-child visits has grown, but large gaps persist among various socioeconomic groups.	To assess and determine if the adherence to recommendations by the American Academy of Pediatrics for WCVs has changed over time.	Medical expenditure panel surveys	N/A	2013	Health Affairs
Zuckerman et al. [26]	Healthy Steps: a case study of innovation in pediatric practice	To evaluate the effectiveness of Healthy Steps as a significant innovation in the delivery of pediatric primary care	Case study	15 pediatric practices	2004	Pediatrics
Coker et al. [27]	Should our well-child care system be redesigned? A national survey of pediatricians	To examine pediatricians' views about whether and how well-child care for children 0 to 5 years of age should be changed	Mail survey	1,000 general pediatricians	2006	Pediatrics
Halloran et al. [28]	Validity of pure-tone hearing screening at well-child visits	To estimate the sensitivity and specificity of pure-tone audiometry hearing screening in the primary care setting	Prospective cohort study	1,061 children	2009	Archives of Pediatrics & Adolescent Medicine
Garg et al. [29]	Addressing social determinants of health at well child care visits: a cluster RCT	To evaluate the effect of a clinic-based screening and referral system (Well Child Care, Evaluation, Community Resources, Advocacy, Referral, Education [WE CARE]) on families’ receipt of community-based resources for unmet basic needs	RCT	336 mothers	2015	Pediatrics
Dinkevich et al. [30]	Evidence based well child care	To evaluate extant guidelines and practices employed in WCCs.	Case study	N/A	2002	BMJ

Quality assessment

The assessment of the included studies’ quality was evaluated using the Joanna Briggs Institute quality assessment tool. The tool scores every publication using the frequency scales that were accorded yes, no, unclear, and not applicable responses. The overall quality score of every study was aptly calculated based on the total amount of positive scores received.

## Review

An overview of the importance of pediatric well-child visits for children aged three to five years

The importance of a pediatric WCV for children aged three to five years should not be underestimated. These visits provide an opportunity for the physician to screen for medical issues, provide anticipatory guidance, and promote good health for the child [6]. They also allow primary care physicians to establish a bond with the parents or caregivers and to prioritize interventions with the strongest evidence for good patient-oriented outcomes, such as family social-economic dynamics, assessment and support, and other health-related goals [6]. Following the U.S. Preventive Services Task Force (USPSTF) and the American Academy of Pediatrics guidelines, immunizations should be updated if necessary, and a one-time vision screening should be carried out between three and five years of age [6]. Additionally, a head-to-toe examination should be performed, including a review of growth [6]. During the visit, the physician can answer any questions the parents or caregivers might have and provide age-appropriate guidance [6]. Furthermore, if any abnormalities are detected, the visit offers the opportunity for further evaluation [6]. Pediatricians are often parents' main formal counseling source for their children's development and education. Their anticipatory guidance can help improve outcomes in various areas such as infant vocal behavior, parenting skills, infant sleep patterns, parental use of discipline, language development, prevention of falls, home accidents, and auto-passenger injuries [7]. The WCV is also a chance to use the CDC-recommended growth charts for assessment and to review parent/caregiver-child interactions [6]. Furthermore, potential signs of abuse should be assessed, and interval growth should be reviewed using appropriate growth charts for height, weight, head circumference, and body mass index [6-7]. Moreover, primary care providers are well-positioned to engage parents and provide referrals to community services during WCVs [8]. Table 2 shows an overview of the main components of a WCV.

**Table 2:** Overview of the main components of a well-child visit.

Visit activity	Overview of detail
Chief complain	The healthcare provider collects any chief complaint the parents may have about their child and also any health update since the last time the child was seen in the clinic (updates like new specialty visits or follow-up from specialty referrals)
Comprehensive assessment	The healthcare provider conducts a thorough physical examination, including height, weight, and head circumference measurements. Blood pressure and heart rate are often checked as well.
Developmental screening	The child's developmental progress is assessed through conversations with parents and caregivers and through observation of the child's interactions and behaviors.
Vision and hearing screening	Basic vision and hearing assessments are performed to identify any potential issues that could affect learning and communication.
Immunizations	The child's vaccination schedule is reviewed and updated as per recommended guidelines. Immunizations are administered to ensure protection against various diseases.
Health education	Parents receive guidance on nutrition, safety measures, oral hygiene, toilet training, and sleep routines. Discussions may also touch on behavior management and discipline strategies.
Screen time and media use	Guidance is provided on appropriate screen time limits and media content for young children, taking into account their cognitive and emotional development.
Counseling	Healthcare providers address parents' concerns or questions about their child's development, behavior, and health.

Benefits of a well-child visit for young children

The benefits of WCVs for young children are numerous [8,9]. WCVs enable pediatricians to review a patient's health history, track a child's growth and development, and provide guidance for parents around topics such as nutrition, sleep, and behavioral development [10,11]. Furthermore, studies have shown that the length of a WCV is linked to the amount of unmet needs experienced by a family [12,13]. This is especially pertinent for children with chronic illnesses or special healthcare needs [12], as pediatric visits are more likely to result in hospital admission than adults [14]. Additionally, WCVs provide an opportunity to discuss prevention, as in the case of childhood obesity [15]. To ensure that WCVs are valuable, there should be an agreement between parents and medical professionals about the visit's goals [7]. Furthermore, physicians should discuss topics such as when to introduce solid foods [6], as this has implications for a child's long-term health. As a result, WCVs can be incredibly beneficial to young children and their families.

Components of a well-child visit for children aged three to five years

This analysis also provided details of the topics discussed during the visits. These topics were classified into seven categories: secondary prevention, primary prevention, health promotion, development, education, and those focused on the parents and family relationship [7]. The study was based on 49 visits to five pediatricians for children aged three to five years [7]. The results showed that the major topics discussed during the visits were illness prevention and education, followed by physical examination and development. Other topics such as health promotion, safety, nutrition, and emotional and mental health were also discussed but in lesser amounts. These results suggest that pediatric providers focus on the primary aspects of a child's physical and mental health, although other topics are often included in the visit. However, this study did not provide specific information about the components of a WCV for children aged three to five years [7]. This means that it is still necessary to understand the specific topics discussed during a WCV to effectively assess a child's health. Therefore, further studies are needed to provide more information about the components of a WCV for children aged three to five years.

Best practices for pediatric well-child visits

A recent study assessed the effectiveness of the Improved Care for Moms and Babies (ICC) model on maternal depression screening and other health behaviors discussed during pediatric WCVs. The study gathered information from mothers who had accompanied their children to WCVs at ages 12 and 24 months [15]. The survey included questions about health history, behaviors, and whether the physician discussed maternal depression, tobacco use, family planning, and folic acid supplementation [16]. The results of the study showed that the ICC model had improved the way maternal depression was being addressed during WCVs. However, best practices for screening, referral processes, and documentation related to PPD screening during WCVs still require further study [17]. This is especially important, as screening for postpartum depression in mothers is recommended during WCVs for young children [17].

Best practices implemented in a well-child visit

Best practices for WCVs are those that provide the most patient-oriented outcomes, such as immunizations, postpartum depression screening, and vision screening [18]. The American Academy of Pediatrics has created guidelines for WCVs, known as the periodicity schedule, and they recommend scheduled WCVs [19]. A study was conducted to determine the impact of the intervention on illness visits between WCVs [10]. The results showed that there were few differences between the two study arms, but a chart review showed that intervention children had fewer illness visits [10]. Pediatric WCVs also provide an opportunity to address psychosocial issues and developmental assessments [20,21], and there is wide variation in practice patterns regarding screening [22]. As far as the duration of WCVs, they are usually short; therefore, strategies for addressing the time devoted to WCVs must be considered [23]. Finally, it is important to understand the best practice of screening, education, and referral processes for addressing psychosocial issues during WCVs [16], as they are a significant part of pediatric office visits [24].

Potential challenges to implementing these best practices in a well-child visit

Additionally, the potential challenges to implementing best practices in a WCV must be acknowledged [18]. For instance, the lack of evidence-based practices can lead to uncertainty about how best to use the limited time available [23]. Furthermore, the Committee on Practice and Ambulatory Medicine of the American Academy of Pediatrics has offered a periodicity schedule to recommend scheduled WCVs [19]. In a study to assess the differences between the interventions and the control arms, it was found that children in the intervention group had fewer illness visits between WCVs than control children [10]. This points to the importance of further understanding best practices for screening, education, and referral during the WCVs [16]. In this regard, research has revealed that 11.6% of the 483 WCVs were with children between 18 and 36 months of age [24]. Moreover, wide variations in practice patterns have been observed with some using a parent questionnaire for all WCVs and some using formal screens only at specific ages [22]. In addition, there is growing interest in understanding the practices of pediatric well-child care internationally [21] and exploring promising strategies for screening during WCVs [17]. Finally, there is a need to consider the ethical and legal dimensions of the boundaries of pediatric care [20].

Guidelines and principles for pediatric well-child visits

The subject of pediatric WCVs has been studied, and a number of guidelines have been developed to ensure that young children receive the best care. Studies have looked into adherence to pediatric WCV recommendations [25], and the standards and principles of Bright Futures [26]. In addition, similarities in approach to child health with periodic visits and anticipatory guidance have been seen abroad [21]. Guidelines recommend scheduled WCVs, detailing the schedule and content of care [19]. This includes preventive care such as guidance directed by a physician [27], and screening tests such as hearing screening [28]. However, further work is needed to optimize well child care [29], and a study found that a schedule with fewer visits had no detrimental effect on child health [30]. Pediatric WCVs are essential for the health and well-being of children, and research has been conducted to ensure that these visits are up-to-date and provide the best possible care.

Implementing guidelines and principles in a well-child visit

Therefore, the implementation of established guidelines and principles for WCVs is necessary [25]. The standards and principles of Bright Futures and the American Academy of Pediatrics [26] are similar in their approach of providing periodic visits and anticipatory guidance [21]. These guidelines and principles are used to define the schedule and content of well-child care [19], which includes guidance directed by a physician and preventive issues [17]. For example, screening tests for children who cannot fully participate are recommended [28]. Furthermore, research has shown that a schedule with fewer visits has no detrimental effect on child health [30]. Even though these articles provide a proof of principle, additional work is necessary to optimize well-child care [20]. Table 3 shows an overview of the basic principles fostering a WCV.

**Table 3:** Basic principles fostering a well-child visit

Principle	Overview
Regular schedule	Well-child visits should be scheduled according to the recommended timeline established by pediatric health organizations, typically occurring annually.
Holistic approach	Healthcare providers should consider the child's physical, cognitive, emotional, and social development as interconnected aspects of their overall well-being.
Parental involvement	Parents and caregivers play a crucial role in the child's health and development. Encouraging open communication and involving parents in decision-making fosters a collaborative approach to care.
Evidence-based practices	Well-child visits should be guided by evidence-based guidelines and practices set forth by reputable pediatric organizations. Healthcare providers should stay updated with the latest research and recommendations.
Cultural sensitivity	Healthcare providers should be culturally sensitive and respectful of diverse family backgrounds, beliefs, and practices. This approach enhances communication and the effectiveness of health interventions.
Developmental surveillance	Providers should remain vigilant for any signs of developmental delays or behavioral concerns. Early identification and intervention can lead to more positive outcomes.
Parent education	Offering clear and accessible information empowers parents to make informed decisions about their child's health, safety, and development.

Potential challenges to implementing these guidelines and principles in a well-child visit

Challenges to implementing effective WCV guidelines and principles arise from multiple areas [25]. For instance, the adherence rate of WCV recommendations may be lower for certain children [26]. Various countries have a similar approach to child health, focusing on periodic visits and anticipatory guidance [21]. The principles of prevention that shape WCV can vary depending on the country [19]. WCV is the most common type of physician visit for children, and it typically involves guidance from a physician [27]. For example, some children may have difficulty participating in screening tests [28]. In addition, further work is needed to optimize WCV, and research has suggested that a schedule with fewer visits may not have a detrimental effect on child health [29]. While there are suggested approaches for WCV programs, these are intended to be illustrative principles rather than specific programs [30]. As such, there are multiple potential challenges to implementing WCV guidelines and principles that must be carefully considered.

Study strengths and limitations

The research conducted in this study possesses several strengths. Firstly, the systematic review follows the PRISMA guidelines, ensuring a rigorous and transparent approach to study selection, data extraction, and synthesis. The search strategy utilized a range of global databases, including Web of Science, EMBASE, PubMed, Cochrane Library, HINARI, and Google Scholar, as well as reference list searches, minimizing the risk of missing relevant studies and enhancing the comprehensiveness of the review. The use of Boolean operators and specific keywords contributes to the thoroughness of the search process; however, the debate surrounding the replication of search results using mesh terms, Boolean combinations, and parentheses for precise literature retrieval stems from concerns about sensitivity and specificity. This debate continues due to factors such as the publication rate, retractions in online scholarly articles, and limitations in search algorithms. Additionally, the focus on studies published within a specific timeframe (2000-2023) enables the inclusion of recent and up-to-date evidence, relevant to the current landscape of acute gout treatment. The evolution and progression of best practices in pediatric well-child clinic visits from 2000 to 2023 have been influenced by advancements in healthcare, changes in technology, evolving clinical guidelines, and a growing understanding of child development and preventive care. The systematic approach to data extraction and quality assessment, including the use of the Joanna Briggs Institute quality assessment tool, enhances the credibility of the findings and the overall reliability of the review. Moreover, the analysis of both monotherapy and combination therapy approaches provides a comprehensive understanding of their respective impacts on serum urate levels, gout symptoms, and overall management. By evaluating various outcomes such as serum urate levels, tophi, gout flare rates, and urinary uric acid, the study contributes a holistic perspective on the effectiveness of the different treatment strategies. Overall, these methodological strengths support the reliability and validity of the conclusions drawn from the research.

However, there are some limitations. Firstly, the scope of included studies is limited to those published between 2000 and 2023, potentially excluding relevant earlier studies that could contribute valuable insights to the topic. Moreover, the inclusion criteria focus on observational studies with specific designs and geographic restrictions, which might omit relevant experimental or international studies that could provide a more comprehensive perspective on the subject matter. Secondly, the diversity in methodologies, patient populations, and study quality across the included studies introduces heterogeneity, which can lead to inconsistencies in findings and complicate direct comparisons between the studies. Despite the use of the Joanna Briggs Institute quality assessment tool, variations in study quality could influence the reliability and robustness of the conclusions drawn from the review. Finally, the search for studies was confined to a selection of global databases and conducted exclusively in English. This approach may introduce publication bias; favoring studies with significant findings and excluding studies published in other languages may limit the representation of findings from non-English-speaking regions, ultimately affecting the generalizability of the conclusions derived from the research.

## Conclusions

Pediatric WCV for children aged three to five years is a vital part of preventive healthcare, addressing physical, emotional, cognitive, and social development. Evidence-based practices ensure comprehensive assessments, vaccinations, developmental screenings, and guidance, thus nurturing a healthy future. These visits benefit immediate and long-term well-being, enhancing individual and community health.

This review emphasizes the importance of WCVs in screening for medical issues, providing guidance, and promoting health. It underlines bonding between physicians, parents, and caregivers, prioritizing evidence-based interventions. Immunizations and socio-dynamic support are highlighted, emphasizing their relevance in WCVs. These visits also offer opportunities for answering parental questions and giving age-appropriate advice. However, challenges exist in implementing guidelines, especially for children with chronic conditions. Primary care providers are essential in engaging parents and linking them to community services, improving outcomes in areas such as vocal behavior, parenting, and language development.

Further research is required to assess individual components' effectiveness in these visits and address implementation challenges, especially for children aged three to five years. In conclusion, policy changes are crucial to underscore the significance of WCVs and ensure that best practices are consistently followed. These changes can lead to better health outcomes, increased vaccination coverage, improved parental education, and reduced healthcare disparities, ultimately benefiting children and communities as a whole.
